# Veterinary students’ proximity to and interpretation of a simulated “aggressive” dog before and after training

**DOI:** 10.1038/s41598-024-53551-w

**Published:** 2024-02-08

**Authors:** James A. Oxley, Georg Meyer, Matthew Butcher, Giuseppe Bellantuono, Andrew Levers, Carri Westgarth

**Affiliations:** 1 Department of Livestock and One Health, Institute of Infection, Veterinary and Ecological Sciences, University of Liverpool, Leahurst Campus, Neston, Cheshire UK; 2 Institute of Digital Engineering and Autonomous Systems, University of Liverpool, Liverpool, UK

**Keywords:** Animal behaviour, Risk factors

## Abstract

Dog “aggression” in the veterinary practice is commonplace. Therefore, student knowledge and education about dog behaviour and the ability to interpret “aggressive” behaviour is important from a human injury prevention and dog welfare perspective. The study aimed to compare first-year veterinary students’ perceived safest proximity to both an “aggressive” and non-reactive simulated dog, both before and after a teaching intervention about canine behaviour and a handling practical. It also examined student confidence and their ability to identify “aggressive” behaviours. Forty first year veterinary students took part in two surveys. Each survey included two videos: one of a simulated dog displaying “aggressive” behaviour, based on the ‘Canine Ladder of Aggression’; and another displaying non-reactive (passive behaviours without reaction to the participants) behaviours. Each video depicted the slow and consistent approach towards the virtual dog within a virtual indoor environment, and participants were asked to press stop if or when they would stop approaching the dog. In the “aggressive” scenario, there was a reduction in the approach-stop time from survey 1 (median = 17.8 s) to survey 2 (median = 15.2 s) in the intervention group (p = 0.018) but not in the control group (p = 0.147). Regarding confidence, there was a significant increase in the self-reported confidence rating relating to a participant’s ability to interpret canine behaviour in both the control (p = 0.011) and intervention (p = 0.003). In conclusion, these results indicate that students using approach-stop videos stayed further away from an “aggressive” virtual dog model if they had undertaken a canine behaviour educational intervention. This novel approach has the potential for further use in teaching and assessment of student knowledge and behaviour which may otherwise be difficult to demonstrate.

## Introduction

Veterinary visits are commonly reported to evoke stress and fear in dogs due to single or multiple stressors (e.g. a previous negative experience, auditory/olfactory stimuli, pain, unfamiliar animals/people, restraint, close contact, etc.)^1–3^. Indeed, such scenarios may result in a fight, flight, or freeze response and in cases of a perceived imminent threat that cannot be avoided (i.e., in a consultation room, being restrained on a table) these options are further reduced to fight and freeze. In such scenarios, behaviours indicative of increasing levels of emotional arousal, often associated with fear, may be displayed such as lip licking, head turn, yawning, paw raise^4^. However, previous research has found that in a survey of Spanish veterinary students^5^ and dog owners^6^ both were less accurate at recognising signals such as yawning, lip licking, turning their head as indicators of stress compared to other signals (e.g., growling, snapping) which were perceived to be stronger indicators of stress in dogs.

Given the subjective and broad nature of the terms “aggressive” and “aggression” it is important that the authors provide a definition^7^. For the purposes of this study the term “aggressive” was defined as a group of behaviours which are displayed by a dog in response to a perceived threat by human or conspecific (also known as social aggression^8^), motivated by a negative affective state (e.g. fear, stress, anxiety) (also known as affective aggression) in order to preserve itself and avoid injury or harm. In a context where a dog cannot escape (e.g. a consultation room or corner of a living room), multiple behavioural signals indicating that the dog is uncomfortable with an approaching perceived threat are initially displayed (e.g. lip lick, yawning, head turn, paw raise, backing away). These signals are displayed in an effort to increase the distance between itself and the perceived threat in order to avoid direct confrontation and injury. If the threat continues, the intensity of the motivational response and behaviours increases to the point where the dog is indicating that they are now direct threat which may include crouching, showing teeth, growling, snapping and finally a bite. The authors refer to the Canine Ladder of Aggression^4^ as a theoretical model example.

Dogs that display “aggressive” behaviour are a concern to veterinary staff and owners due to the potential for dogs to bite causing minor to severe injury and in rare cases death^9^. Such incidents may result in time off work, treatment/surgery, infection and/or zoonotic disease transmission all of which have potential legal and financial implications^9–11^. Of course, fear itself is a welfare concern for the individual animal and efforts need to be considered to address this e.g., veterinary practice layout optimised^12^ and low-stress handling^13^. In addition, the initial purpose of the veterinary visit could also be due to a pre-existing canine behaviour problem^14^.

Occupational injuries to veterinarians and other veterinary staff, such as animal bites, are commonplace and have even been referred to as being “part of the job”^15^. Nienhaus et al.^16^ found that, in Germany, veterinary staff were 2.9 times more likely to suffer a work-related injury in comparison to medical staff. Furthermore, they also found that over half (59.7%; 1077/1805) of work-related injuries to veterinary staff were due to animals causing cuts, bites or scratches. Injuries to veterinarians and veterinary staff due to ‘bites’, ‘dog and cat bites’ or ‘bites, scratches or cuts’ have been reported as one of the most common within veterinary practices in Australia^17,18^, USA^19^, India^20^ and Canada^21^. The British Veterinary Association^22^ found that 62% (292/474) of veterinarians surveyed suffered a work-related injury in the past twelve months. Of the companion animal vets (251/292), the majority (75%; 188/251) said they had been bitten by an animal, the second highest injury type reported among companion animal veterinarians^22^.

Mannion et al.^23^ found that 87% of veterinary students thought it likely that they would be bitten by a dog during their veterinary career. Indeed, Landercasper et al.^19^ found that 92.3% of veterinarians from the US states of Minnesota and Wisconsin had received a dog bite during their veterinary career. More recently, Fritschi et al*.*^24^ found that 48% of 2718 Australian veterinarians received a dog bite or scratch resulting in a puncture to the skin within the past 12 months. Interestingly, of these, veterinarians who graduated between 1990 and 2000 were 2.55 times (CI 95% 1.86–3.50) more likely to be bitten in the last 12 months compared to veterinarians that had graduated between 1960 and 1969. It is possible that this discrepancy is related to the relative clinical experience of the veterinarians which may be a factor in dog bite prevention in a clinical setting.

Previously, in the UK, according to Mannion et al.^23^ *“most veterinary students and veterinary school curricula do not have any formal teaching in the assessment and recognition of dog behaviour or of the signs of canine aggression”* (p. 536). However, it is unclear if this remains to be the case in the UK. Recently, Kogan et al.^25^ surveyed US veterinarians and found that despite almost all (99.6%) participants seeing behavioural cases in practice, less than half (43%) of veterinarians felt they received enough animal behaviour training during veterinary school whilst 39% and 18% stated they received a few hours and none respectively. Furthermore, veterinarians most frequently reported being uncomfortable or very uncomfortable when dealing with behavioural problems including human-directed “aggression” and ‘fear biting’.

Therefore, the importance of investigating the effectiveness of the teaching and assessment of veterinary students’ knowledge and understanding of canine behaviour, including both normal and abnormal behaviour, is threefold. Firstly, accurate knowledge and the ability to differentiate between normal and abnormal canine behaviour is important in identifying any potential health and welfare-related factors associated with illness or poor health e.g. “aggression” due to pain^26^. Secondly, as with the public, appropriate knowledge of canine behaviour is likely to indicate if the dog wants to be approached and the veterinarian’s behaviour and method of approach and handling can be adjusted accordingly^26^ and this is important for the safety of the veterinary staff, owners and dogs. Thirdly, canine behavioural knowledge is fundamental in the prevention, diagnosis, and appropriate treatment (or referral), of behavioural problems^27^.

The present study aimed to use an online survey with videos to:

1. Assess veterinary students perceived safest proximity to approaching an “aggressive” and non-reactive simulated dog.
  *Hypothesis 1* Veterinary students will move significantly closer to the non-reactive dog in comparison to the dog displaying “aggressive” behaviour.
2. Compare the difference in students’ perceived safest proximity to both the “aggressive” and non-reactive simulated dog, prior to and after a teaching intervention about canine behaviour and handling practical.
  *Hypothesis 2* Veterinary students will stay further away from the dog displaying “aggressive” behaviour in the post-intervention survey compared to the pre-intervention survey.
3. Compare the difference in perceived safest proximity between the intervention group (see aim 2) and a control group that has not received the teaching intervention.
  *Hypothesis 3a* The control groups will get closer to both the “aggressive” and non-reactive dogs compared to the intervention group.
4. Assess participants’ recognition and interpretation of behaviours based on the Canine Ladder of Aggression, prior to and after the teaching intervention and compare to a control group assessed twice pre-intervention.
  *Hypothesis 4* Students will be less likely to recognise the signals displayed by the virtual dog early in the “aggressive” scenario (e.g. lip licking, yawning) than those displayed by the dog later in the sequence of behaviour (e.g. showing teeth).
5. Examine the recognition and the perceived safest proximity to the dog by the student’s related experience.
  *Hypothesis 5* Those who have been previously bitten by a dog and/or have less frequent contact will show a reduction in approach-stop time in comparison to those who have not been previously bitten and/or have more contact with dogs.
6. Assess the realism of the appearance and presence of the virtual dog model.
  *Hypothesis 6* The majority of participants will agree that the dog model’s appearance and behaviour are realistic.

## Results

**Figure 1:**
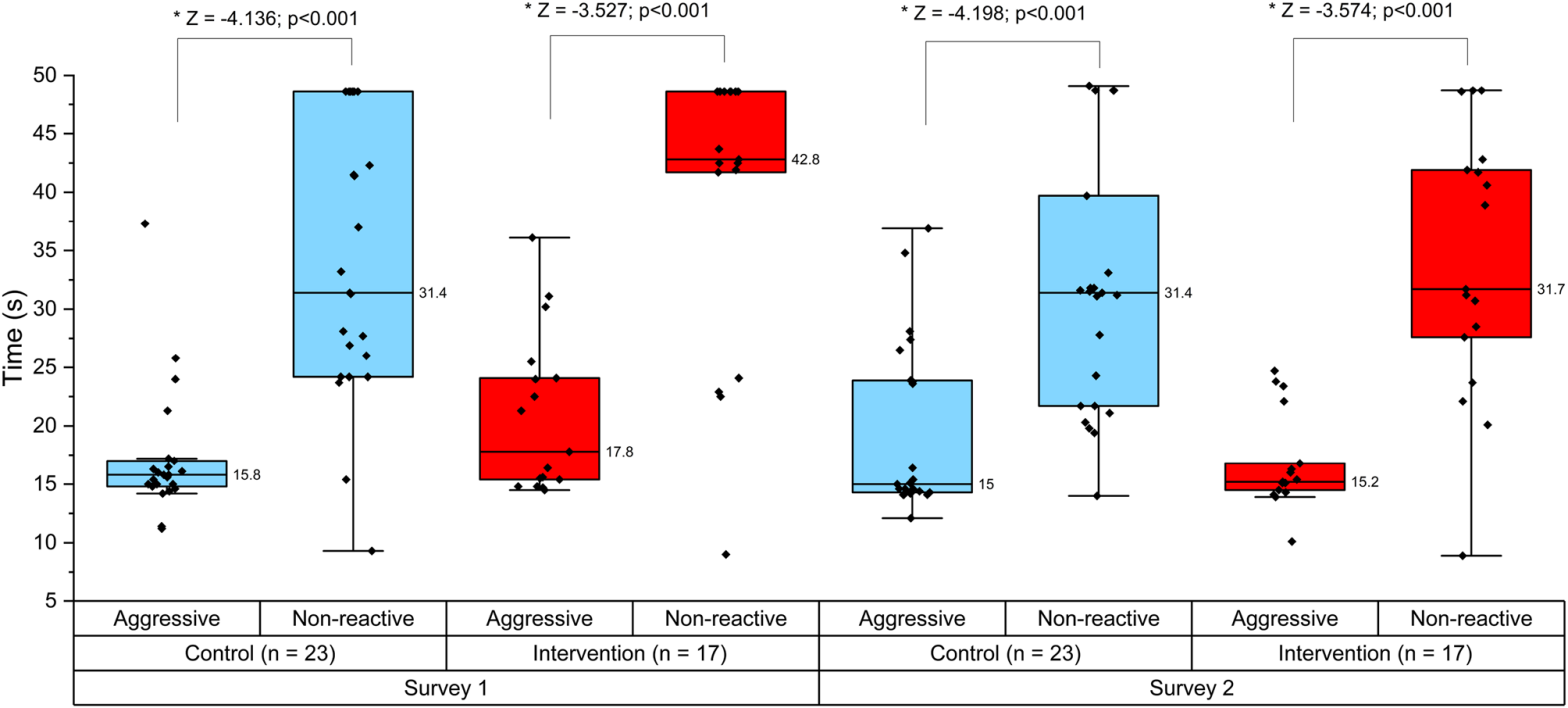
All approach-stop tasks for control and intervention groups in survey 1 and 2 and within group comparisons between the “aggressive” and non-reactive scenarios. Whiskers indicate values that are < 1.5 times the interquartile range.

Overall, a total of 40 out of the 209 veterinary students (19.1%), completed both the survey 1 and 2. This included a control group (n = 23) and intervention group (n = 17) (Fig. 1). Eight of the control group also completed the third survey post-intervention wait-list, which when added to the intervention group findings resulted in 25 participants with data for pre-post intervention.

### Participant demographics

Most participants were female (92.5%), which was a higher proportion of females compared to the overall class (84.2%; 176/209). There was no difference in age between the control and intervention groups (p = 0.386) (Table 1). Most participants currently owned a dog and had owned a dog for nine years or longer. Seventy per cent of participants currently or previously worked with dogs (either voluntarily and/or paid) (Table 1). Regarding dog bites causing bruising or puncture to the skin, 35% of participants in both the control and intervention group reported having been previously bitten.

**Table 1:** Participants’ demographics and dog-related experience.

	Control (*n* = 23) n (%)	Intervention (*n* = 17) n (%)	Total (*n* = 40) n (%)
Age-mean years (range)	19.9 (18–25)	20.7 (18–39)	20.2 (18–39)
Gender			
Female	22 (95.7)	15 (88.2)	37 (92.5)
Male	1 (4.3)	2 (11.8)	3 (7.5)
Dog ownership			
No	2 (8.7)	4 (23.5)	6 (15.0)
Yes, previously	3 (13.0)	1 (5.9)	4 (10.0)
Yes, currently	18 (78.3)	12 (70.6)	30 (75.0)
Length of dog ownership			
9 years or longer	15 (71.4)	11 (84.6)	26 (76.5)
7≤9 years	1 (4.8)	0	1 (2.9)
5≤7 years	1 (4.8)	1 (7.7)	2 (5.9)
3≤5 years	2 (9.5)	0	2 (5.9)
1≤3 years	1 (4.8)	1 (7.7)	2 (5.9)
<1 year	1 (4.8)	0	1 (2.9)
Frequency of contact with dogs (other than owned dogs)			
At least once a day	3 (13.0)	1 (5.9)	4 (10.0)
Several time a week	10 (43.5)	6 (35.3)	16 (40.0)
Several times a month	7 (30.4)	6 (35.3)	13 (32.5)
Less than once a month	3 (13.0)	4 (23.5)	7 (17.5)
Current/previous work with dogs			
Yes	16 (69.6)	12 (70.6)	28 (70.0)
No	7 (30.4)	5 (29.4)	12 (30.0)
Current/previous hobby with dogs			
Yes	3 (13.0)	3 (17.7)	6 (15.0)
No	20 (87.0)	14 (82.3)	34 (85.0)
Previously bitten (causing at least bruising or skin puncture)			
Yes	8 (34.8)	6 (35.3)	14 (35.0)
No	15 (65.2)	11 (64.7)	26 (65.0)

### Canine behaviour

Two participants (5.0%) stated they had undertaken a formal or informal canine behaviour course before their veterinary degree. Between surveys 1 and 2 there was an increase in participants who stated they had been introduced to the concept of canine behaviour during their veterinary degree by 70.6% in the intervention group (p < 0.001) but only 13% in the control group (p = 0.250) (Table 2). In survey 1, 64.7% of the intervention group stated they had ‘not at all’ been provided with formal teaching in canine behaviour compared to 0% in survey 2 (p = 0.003). In contrast, there was a small (not significant) decrease (17.4%) in those reporting receiving no formal teaching in canine behaviour in the control group between surveys 1 (78.3%) and 2 (60.9%) (p = 0.59) (Table 2).

**Table 2:** Participants’ responses to questions relating to introduction to the concept of canine behaviour and the formal teaching of canine behaviour so far.

	Control			Intervention		
So far, during your veterinary degree, have you been introduced to the concept of canine behaviour?	Survey 1 n (%) (n = 23)	Survey 2 n (%) (n = 23)	p value	Survey 1 n (%) (n = 17)	Survey 2 n (%) (n = 17)	p value
Yes	8 (34.8)	11 (47.8)	0.250*	4 (23.5)	16 (94.1)	< 0.001*
No	14 (60.9)	12 (52.2)		12 (70.6)	1 (5.9)	
I don’t know	1 (4.3)	0		1 (5.9)	0	
So far, during your veterinary degree, approximately how much formal teaching in canine behaviour have you received?						
Not at all	18 (78.3)	14 (60.9)	0.221**	11 (64.7)	0	0.003**
Half a day or less	5 (21.7)	8 (34.8)		6 (35.3)	9 (52.9)	
1 day	0	1 (4.3)		0	5 (29.4)	
≥ 2 days	0	0		0	3 (17.7)	

*McNemar’s test using yes (1) and no (0) values only.

**McNemar’s test using formal teaching (1) or no formal teaching (Not at all) (0).

### Dog related statements

Participants were asked to complete the six dog-related statements on both the survey 1 and 2. In both surveys, over 90% of all participants agreed with the statements regarding enjoying and feeling relaxed in the presence of most dogs and perceived ability to recognise ‘relaxed’, ‘aggressive’ and/or ‘scared/fearful’ behaviour (Supplementary material 2).

### Perceived virtual dog appearance and behaviour

Most participants (≥ 80%) agreed with the two statements stating that the appearance and behaviour of the dog was similar to that of a real dog in both the non-reactive and “aggressive” videos (Supplementary material 3).

### Self-rated confidence

There was a significant increase in the self-reported confidence rating relating to a respondent’s ability to interpret canine behaviour between the survey 1 (pre-intervention 1) and 2 (pre-intervention 2) in both the control group (Z = − 2.546; p = 0.011; *r* = 0.4) and the intervention group (Z = − 2.972; p = 0.003; *r* = 0.5) (Table 3 and Supplementary material 4). A significant increase in confidence ratings was also evident between survey 1 and survey 2 of the combined intervention group (Z = − 3.252, p = 0.001; *r* = 0.7). Thus, there was no difference in the change in scores (between survey 1 and 2) between the control and intervention group (p = 0.516) as all groups increased in confidence (Table 3).

**Table 3:** Pre-post change in confidence ratings and approach stop times within the control and intervention groups.

Measure	Median (control)	Median (intervention)	Control/int. comparison of change
Confidence			
Pre-intervention 1	3 (2 to 5)	3 (1 to 4)	
Pre-intervention 2	4 (3 to 4)	4 (3 to 5)	
Change	0.5 (− 1.0 to 3.0)	1 (0 to 2)	
P value	**0.011**	**0.003**	0.516
Approach-stop task (time)			
Non-reactive 1	31.4 (9.3 to 48.6)	42.8 (9.0 to 48.6)	
Non-reactive 2	31.4 (14.0 to 48.6)	31.7 (8.9 to 48.6)	
Change	0 (− 19.8 to 11.0)	− 0.1 (− 28.5 to 17.7)	
P value	0.376	0.156	0.401
“Aggressive” 1	15.8 (11.2 to 37.3)	17.8 (14.5 to 36.1)	
“Aggressive” 2	15.0 (12.1 to 36.9)	15.2 (10.1 to 24.7)	
Change	− 0.2 (− 5.90 to 12.0)	− 2.1 (− 6.0 to 20.9)	
P value	0.417	**0.018**	**0.012**

Significant values are in bold

This table does not include the combined intervention group

### Approach-stop task

#### Within-group comparison: non-reactive compared to “aggressive” scenario

In both the control and intervention group, participants spent significantly longer (p < 0.001) approaching the non-reactive dog (i.e. they got closer) compared to the “aggressive” dog (Fig. 1) (despite the shorter length of the video). This was also the case for the Survey 3 (combined intervention group) for both the pre (p < 0.001) and post survey (p < 0.001).

#### Within-group comparison: pre-compared to post-intervention

In the “aggressive” scenario, there was a reduction in the approach-stop time from survey 1 (median = 17.8 s) to survey 2 (median = 15.2 s) in the intervention group (Z =− 2.367; p = 0.018; *r* = 0.4) and the combined intervention group (survey 1 (median 15.8 s), survey 2/3 (15.1 s); Z = − 2.153; p = 0.031; *r* = 0.4), but not in the control group (p = 0.417) (Table 3). There was no difference in approach stop times between survey 1 and 2 in the non-reactive scenario in the control (p = 0.376), intervention group (p = 0.156) (Table 3) or combined intervention group (p = 0.362).

#### Between-group comparison: change pre-post intervention

There was no evidence of a significant difference in pre-post change in approach-stop times between control and intervention groups for the non-reactive scenario (p = 0.401). In the “aggressive” scenario there was a significantly larger pre-post change in approach-stop time in the intervention (median: − 2.1 s) group compared to the control group (median − 0.2 s) (Z = − 2.491; p = 0.012) (Table 3).

### Approach-stop time and dog related experience

There was no evidence of a difference in approach-stop times towards the dog displaying “aggressive” behaviour between individuals that had and had not been previously bitten in both the control (survey 1: p = 0.286; survey 2: p = 0.582) and intervention group (survey 1: p = 0.651; survey 2: p = 0.131). There was also no evidence of a difference between approach-stop times based on frequency of contact with dogs in the control (survey 1: p = 0.074; survey 2: p = 0.065) and intervention group (survey 1: p = 0.794; survey 2: p = 0.200).

### Timing of approach-stop task in relation to canine behaviours displayed

Most participants activated the stop button when the dog’s behaviour was within zone 1 in both the control [survey 1: 95.7% (22/23); survey 2: 87.0% (20/23) (Z = − 1.342; p = 0.180)] and the intervention group [survey 1: 82.4%; (14/17); survey 2: 100% (17/17) (Z = − 1.633; p = 0.102)]. The highest zone of behavioural responses that any participant reached before activating the stop button was level 6 out of the 8 levels. The median stopping time for participants who stopped in zone 1 were 15.2 s (standing and moving head upwards) post-intervention (survey 2) compared to 16.0 s (dog standing still looking at the user) pre-intervention (survey 1). In the control group median stopping time for participants who stopped in zone 1 in survey 2 was 14.6 s (dog takes a step backwards) compared to 15.2 s (dog takes a step backwards and raises its head) in survey 1.

### Recognition and interpretation of behaviours

Of the participants in both the control (n = 20) and intervention (n = 16) groups that completed the behavioural interpretation section in both surveys, the vast majority (> 90%) stated that the emotions the dog was showing in the video were ‘anxious’ and ‘scared/fearful’ in both survey 1 and 2 (Table 4). Most participants (≥ 80%) in both control and intervention groups noticed growling and/or barking (Table 4).

**Table 4:** Types of emotions and vocalisations noticed by participants during the aggressive full-length video.

	Survey 1 control n (%)	Survey 2 control n (%)	Survey 1 int n (%)	Survey 2 int n (%)
What emotions do you think the dog was showing in the last video?				
Anxious	20 (100)	19 (95)	15 (93.8)	16 (100)
Scared/fearful	20 (100)	20 (100)	15 (93.8)	15 (93.8)
Anger	8 (40)	7 (35)	8 (50)	8 (50)
Sad/upset	5 (25)	5 (25)	5 (31.3)	2 (12.5)
Shy	4 (20)	3 (15)	3 (18.8)	4 (25)
Calm/relaxed	1 (5)	2 (10)	1 (6.3)	3 (18.8)
Total	20	20	16	16
Missing	3	3	1	1
Did you notice any dog related sounds or vocalisations?				
Yes	20 (95.2)	20 (95.2)	16 (100)	15 (93.8)
No	1 (4.8)	1 (4.8)	0 (0)	1 (6.2)
Total	21 (100)	21 (100)	16 (100)	16 (100)
Missing	2	2	1	1
If yes, explain what [sounds or vocalisation] you noticed? (Multiple choice)				
Barking	20 (100)	18 (90)	14 (87.5)	12 (80)
Growling	17 (85)	19 (95)	15 (93.8)	15 (93.8)
Yawn/whining/whimpering	11 (55)	12 (60)	4 (25)	6 (40)
Panting	8 (40)	4 (20)	7 (44)	9 (60)
Snarling	3 (15)	3 (15)	3 (18.8)	3 (18.8)
Other*	2 (10)	2 (10)	3 (18.8)	2 (13.3)
Total	20	20	16	16
Missing	3	3	1	1

*Other: Lip licking (4), Yelp (2), Snapping (1), Grumbling (1), Squeaking (1).

*Only participants were included that completed the questions for both survey 1 and survey 2 in the control (n = 20) and intervention (n = 16) group.

All participants stated that they noticed the behaviours included in the higher time zones such as the dog moving backwards and showing their teeth (supplementary material 5). In the control group, there was an increase, although not significant, in the reported recognition of three behaviours between survey 1 and survey 2 (including lip lick (75% to 95%), paw raise (65% to 90%) and head turn (75% to 95%). In the intervention group, the lip lick behaviour recognition increased from 50% to 87.5% pre and post intervention respectively (p = 0.031) (supplementary material 5).

In the context of the full video footage, the perceived meaning of lip lick, yawn and paw raise were for the majority of participants referred to as negative arousal or emotion (see supplementary material 6, 7 and 8). In the intervention group only, the term anxious to describe the lip lick increased from 25% (4/16) in the pre-test (survey 1) to 50% (8/16) in the post test (survey 2) (p = 0.125).

### Presence

There was no evidence of a difference between median presence ratings across both the control and intervention groups and within group pre and post surveys (supplementary material 9).

## Discussion

This study examined the effectiveness of a series of lectures and a practical lesson involving canine behaviour interpretation for undergraduate veterinary students, using an intervention and control group. It found that students from the intervention group reduced their approach-stop time (i.e. stopped further away) from a virtual dog showing “aggressive” behaviours post-intervention in comparison to pre-intervention. In contrast, the control group showed no evidence of change. Interestingly, there was no evidence of variation in approach-stop distance depending on whether participants had previously worked with dogs, frequently had contact with dogs, or previously had been bitten by dogs, however sample sizes for these analyses were small and there may not have been power to detect.

Participant confidence in the interpretation of dog behaviour increased between survey 1 and survey 2, however, this was seen in both the control and intervention groups, so this may not be due to the intervention of the canine behaviour lessons. Similarly in the control group recognition of three behaviours increased between surveys 1 and 2. It could be argued that simply repeating the survey (i.e. practice effect) could increase participant confidence and behaviour recognition in the second survey especially as the same videos were used in both surveys 1 and 2. This demonstrates the importance of using a control group comparison in studies to identify the effect of an intervention on an assessment of behaviour in this way.

Barking and growling were the most recognised vocalisations after watching the full-length “aggressive” video. This is consistent with previous research, for example, Menor-Campos et al.^5^ found that excessive barking was most categorised as strongly related to behavioural stress indicators in fourth-year veterinary students.

Over 90% of participants stated that the emotion the dog was showing was anxious, scared or fearful. These emotions indicate that the participants were aware that the dog was showing some form of negative valence and high degrees of arousal^28^. However, the form of “aggression” in the present study was specifically defensive aggression often noted as a result of fear to a perceived threat^29^. This may highlight that there is a lack of knowledge regarding specific terminology and if or how they differ in dog behaviour. For example, although there is likely to be some overlap, anxiety generally refers to the anticipation of a threat, that is not currently present, due to uncertainty^30^. Fear on the other hand is the reaction to an actual or perceived threat which is present in the environment^30,31^. Further research would be useful to explore veterinary students’ understanding of their perceived meanings and differences between such terms and if or how these could be applied to help understand defensive aggression in dogs.

In the approach-stop task, the majority of participants stopped in level 1 during which the dog was displaying signals of response to a potential perceived threat including taking two steps backwards (occurred between 13.3 and 15.1 s) followed by standing and staring directly at the user with a closed mouth and raised head (occurred between 15.1 and –20.3 s). Large body movements have been previously identified when describing “aggressive” or fearful behaviours in dogs^32^. A simple explanation could be as the dog is taking two steps backwards this could be interpreted as the dog trying to avoid or move away from the oncoming threat.

It was encouraging to note that the majority of participants agreed that even a 2D virtual dog model had similar behaviour and appearance to that of a real dog. This is likely due to multiple factors including the quality of the animation, and the behaviours displayed were based on realistic dog behaviour and reviewed by canine behavioural experts. This highlights the importance of collaboration between animation developers and dog behavioural experts^33^.

In this case, the practicalities of the assessments (which had to be virtual due to COVID-19, and not impact teaching time) limited the study design. In future, the surveys could be made compulsory as part of the lesson to ensure a larger sample size and avoid self-selection bias, for example, veterinary students with experience as a dog owner or with a particular interest in, or knowledge about, dogs may be more inclined to complete the questionnaire. It is important to note that the question relating to confidence uses the broad term canine behaviour. It is possible that the mention of canine behaviour or “aggression” was briefly discussed during the curriculum outside of the specific interventions we were assessing. While the control group had not completed the formal teaching before survey 2, these students could have conducted independent research on the topic and thus increased their knowledge. It is also possible that after the first survey students could have discussed the survey and influenced each other’s opinion or answers for survey 2. In future research, more detailed questions could ask about students’ formal and informal methods of acquiring knowledge on dog “aggression”. To avoid the potential for participants acquiring dog behavioural knowledge between surveys that are several weeks apart, it would be useful for the surveys to be administered directly before and after the taught and practical session and a final follow-up several weeks later to ascertain knowledge retention.

This study involved multiple teaching intervention components. The questions asked in the surveys were based upon the Canine Ladder of Aggression terminology. Participants were offered options for the meaning of different behavioural signs based on that teaching. Various aspects of the Companion Animal Welfare and HACS course teaching discussed different terminology and other options for answering the questionnaire were not available. In future studies, it could be beneficial to streamline teaching approaches and have questions relating to all approaches that the students are exposed to during teaching included in the survey.

The majority of participants had previously or currently owned, worked or had a hobby with dogs. This could have affected their ability to interpret dog behaviour and select a stop distance. Although similar to our findings, previous research found no relationship between pre-class animal behaviour knowledge and pet ownership in first year veterinary students^27^, attachment and bond of dog-owning students should be considered in future research as Menor-campos et al.^5^ recently found that attachment level was associated with higher identification of subtle dog stress signs (e.g., looking elsewhere and turning head) in veterinary students.

Furthermore, this study focuses on first year students only and in one of nine university veterinary schools in the UK at the time^34^. Further research could expand to all veterinary students (years 1–5), qualified veterinarians and veterinary staff such as nurses, and technicians. Practical methods and course content for veterinary professional students will likely vary which could be assessed through a multi-site study.

Only three males completed both the survey 1 and 2 and therefore comparisons between gender were unable to be made. Previous research does highlight a similar gender bias in veterinary students’ response rate (i.e. > 90% female)^35^. However, gender comparison is of importance as male vets have been reported to be more likely to suffer bite injuries in comparison to females^24^ and males have higher hospital admission rates in relation to dog bite injuries^36^. However, Menor-Campos et al.^5^ stated that in an online survey, there were no differences between male and female veterinary students in their ability to recognise signs of stress in dogs.

In this study of veterinary students, two specific additions could be made. Firstly, additional questions to encourage the participant to think and discuss the possible options to deal with the dog’s behaviour through interactive questions and options (e.g., attempt to restrain the dog, feed the dog treats). Secondly, additional virtual environments could be developed to resemble a veterinary practice or kennels allowing the student to undertake the video tasks in multiple environment types.

Finally, the online virtual approach stop task had limitations compared to actual virtual reality, including the user could not move backwards (away from the dog) after stopping. There was also no option for the user to remain stationary and offer for the dog to approach, as many may choose to do to reduce the risk of confrontation. Further development of the videos and the ability for the user to adjust the distance may be of use. In addition, comparisons between virtual reality and online surveys could be made to compare levels of immersion and realism and assess the potential difference in behaviour recognition and approach-stop distances. Given the limitations, caution is needed when interpreting the results.

Most veterinary students agreed that the behaviour and appearance of the video of a virtual dog was similar to that of a real dog in both the non-reactive and “aggressive” videos.

Between survey 1 and 2 there was evidence of a reduction in approach-stop time in the “aggressive” scenario for the intervention group but not the control group. This suggests that the veterinary students receiving canine behaviour and handling training did practice safer behaviour by reducing the approach-stop time when participating in the “aggressive” dog scenario. Furthermore, the approach-stop task appears to be a viable way to assess learning outcomes and potential behaviour change.

## Method

This study was approved by the University of Liverpool’s Veterinary Research Ethics Committee (VREC1042/VREC1042a) and all research in this study was conducted in accordance with the Declaration of Helsinki. All participants took part voluntarily and all provided informed consent at the start of each survey.

### Survey

Online questionnaires were developed and then reviewed by two veterinary behaviourists; both were qualified veterinary surgeons (RCVS) and Certified Clinical Animal Behaviourists, and one was a European Veterinary Specialist in Behavioural Medicine. As a result, the questionnaire was subsequently updated, and the final version was approved by the year lead for first-year Bachelor of Veterinary Science (BVSc). The survey was hosted by the online software Gorilla Experiment Builder (www.gorilla.sc) ^37^. The online survey was completed by participants voluntarily as this was not a compulsory part of their degree.

The survey was presented multiple times to participants over the study period. At the start of the first survey, an information page covered the purpose of the research, voluntary participation, inclusion criteria (first-year veterinary students), ethical approval and researcher contact information. It was recommended that the survey be taken on a PC or laptop with the sound on at a normal level.

The survey included four sections. Section one part A was only conducted in the first survey and included questions relating to veterinary student demographics (gender, age, education level), dog ownership (previous/current and length of ownership), dog-related experience (contact with non-owned dogs, dog-related job or hobby), previous experience of being bitten (causing bruising or puncture to the skin^38^), previous canine behaviour courses taken. Both the first and second surveys then enquired in section one part B about the amount of formal teaching students had received about canine behaviour so far during the BVSc degree, confidence in their ability to interpret canine behaviour (ordinal scale; 1 = not at all confident to 5 = very confident), and agreement to six statements (five-point Likert scale; strongly agree to strongly disagree) including three questions related to an individual’s feelings in the presence of a dog, and three questions about their perceived ability to recognise “aggressive”, relaxed and scared/fearful dog behaviours.

Section two included two video tasks consisting of an “aggressive” and non-reactive dog model. Each participant was shown both videos but in a random order so that potential order effects were taken into account. The reactive dog model and behaviours were based on the theoretical model called the ‘Canine Ladder of Aggression’^4^ (see supplementary 1) and the environment (an indoor living room) was the same as those used in a previously published virtual reality task^39^. The “aggressive” dog model consisted of 6 different levels. At level 0 the dog was lying down until user movement started in which case the dog stood up. The behavioural signs from the dog in the scenarios labelled as “aggressive” were categorised into zones 1, 5, 6, 7, 8 according to how far into the behavioural sequence, in time, they occurred. Level 2, 3 and 4 did not occur as these levels were only activated if the user was stationary and therefore was not applicable in this video due to the continuous movement. Behaviours in zone one included signs such as the dog taking a step backwards, standing and moving head upwards and yawning and lip licking. In contrast zone 8 included signs such as the dog crouching, baring teeth, eyes widened with whites of the eye visible, lip licking and growling (see supplementary material 1 for levels and behaviours). In the non-reactive video the dog behaviour included standing up looking left and right sniffing the ground, sitting with mouth open and a relaxed tail, and taking a step forward (see Oxley et al.^39^). The non-reactive dog video was shorter in length (48.6 s) compared to the “aggressive” dog (56.6 s), as the “aggressive” dog moves backwards, and the non-reactive dog does not. The purpose of the task in each video was to ascertain the proximity participants get to the dog model.

In this study, the movement in the videos was not controlled by the user and consisted of a consistent speed from the start to the end of the videos. Participants were instructed to *“Once* *the* *video* *starts*, *press the STOP button at the point that you would stop, IF you would stop approaching the dog before the end of the video. Please click the video to start. A countdown to the video will occur”.*

Section three involved asking participants to watch a full video of the “aggressive” dog. Questions were then asked if they noticed any vocalisations and perceived emotions of the dog, what they noticed in general about the dog’s behaviour, and if they noticed specific behaviours (lip lick, yawning, paw raise, head turn, backing away and showing teeth) and their perceived meaning for each behaviour. Section four included the Igroup Presence Questionnaire which consisted of 14 questions^40^. Once completed, a debrief information sheet was displayed informing them of the study information, how data will be used and study contact information.

### Participants

A total of 209 first-year veterinary students were enrolled on the BVSc degree (academic year: 2020–2021) at the University of Liverpool. At the start of the degree, students were allocated into seven groups, six with 30 students and one with 29, for their practical sessions.

### Interventions

There were two main educational interventions which were included as part of this study and were part of the BVSc course. The first intervention included a Companion Animal Welfare lesson taught to a single class, including all students, on the 15th January 2021. Attendance was not compulsory, and recordings of the lecture was available for students. The lesson included relevant topics relating to indicators of fearful and aggressive behaviour in dogs. Specific reference and descriptions were made to the Canine Ladder of Aggression and YouTube videos were used to discuss body language of a fearful dog including early signs.

The second intervention included a handling of animals course with teaching specific to dogs and cats, which all students were required to attend. This included multiple online lecture (due to COVID) and further resources to read/watch, in addition to a 2 h in-person handling practical. A component of the teaching materials involved a lecture delivered by a veterinary behaviourist focused on recognising the range of independent behavioural responses to protective (negative) emotions in dogs and discussing how this leads to an individualised approach to dogs in a clinical context. The dog and cat practical handling session consisted of a variety of areas including small animal behaviour (i.e. signals indicating high vs low arousal, recognition of behavioural signs: e.g., appeasement), capture and control (e.g. dealing with a fearful animal in a confined space), handling and restraint (e.g. restraint of fearful animal), restraint for common procedures. Additional notes were provided online covering small animal handling relevant to dog behaviour and again covering the Canine Ladder of Aggression, assessment of dog body language, and behaviour recognition of a dog that is fearful and stressed.

### Procedure

**Figure 2:**
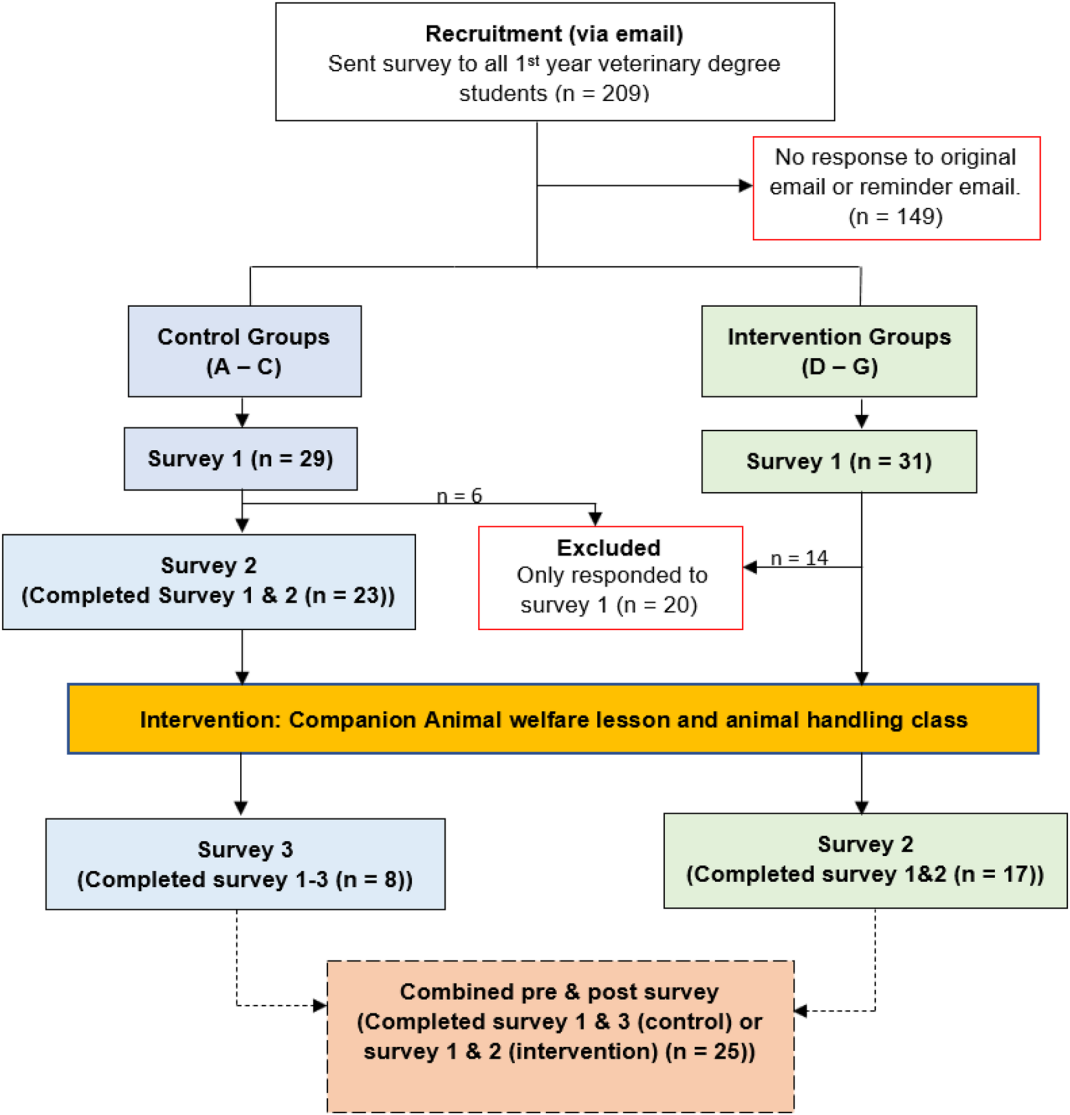
Recruitment and participation flowchart of veterinary students. Both the control (Pre intervention survey 1 and 2) and intervention group (pre-intervention survey 1 and post-intervention survey 2) were asked to complete two surveys. However given the low response rate for the intervention group (n = 17), the control group was invited to complete a third survey after they had attended their practical/teaching session meaning that this resulted in a ‘combined group’ sample (n = 25) (i.e. control (survey 3; n = 8) and the intervention group (n = 17)).

Students were allocated into two study groups based on the time of their practical handling teaching sessions that occurred for each group. Both the control (n = 90) and intervention (n = 119) groups were sent the first survey by email between 18th and 19th December 2020 and a reminder on the 4th January 2021, before any teaching sessions occurred (Fig. 2). The second survey (Survey 2) for control group was sent out on the 11th of January and a reminder on the 14th January 2021 which was again before any teaching sessions had taken place. The intervention group was sent survey 2 the week after their final practical teaching session which occurred between mid-February and early March 2021. As the survey was entirely voluntary, to maximise completion a follow-up reminder between 1 and 2 weeks after the invitation was sent. Given the low response rate for the intervention group, the control group were invited to complete a third survey (post-intervention) on the 19th February 2021, after they had attended their practical/teaching session, and a final reminder was sent on the 9th March 2021. The control group participants who completed survey 1, 2, the intervention and survey 3 for the control group (n = 8) were added to the intervention group for sub-analyses to increase the sample size and are hereafter referred as the ‘combined intervention group’ (Fig. 2).

### Statistical analysis

All data were exported from the online survey software Gorilla Intervention Builder into Excel. Statistical analysis was conducted in Microsoft Excel (Microsoft Office 365, Microsoft Corp.) and SPSS (version 27, IBM, Armonk, NY: IBM Corp). Descriptive statistics were summarised and presented in percentages. For both ordinal (confidence scale) and continuous (time taken to stop the videos) data the Wilcoxon signed rank test was used for within group (pre-post surveys) and the Mann–Whitney *U* tests for between groups (control versus intervention) comparisons. Pre-post change per group was calculated by subtracting pre-survey approach stop time from the post-survey approach stop time and the size effects calculated (z/√N)^41^. A Kruskal–Wallis test was used to assess if there was a difference in approach stop time based on the frequency of contact with dogs. Recognition of behaviours (e.g. lip lick = yes or no) between survey 1 and survey 2 was analysed for both the control and intervention group using McNemar’s test. A p-value of < 0.05 was considered significant. Open-ended questions related to the meaning of dog behaviours were coded into themes using the qualitative statistical software Nvivo Pro (Version 12, 2020, QSR International Pty Ltd.). Boxplots were created in the software Origin (2020b, OriginLab Corporation, Northampton, MA, USA).

### Supplementary Information

Supplementary Tables.

## Data Availability

The datasets generated during and/or analysed during the current study are available from the corresponding author on reasonable request.
