# Supplementary material for: Veterinary students’ proximity to and interpretation of a simulated “aggressive” dog before and after training
Source: Sci Rep. 2024 Feb 8;14:3209. doi: 10.1038/s41598-024-53551-w (PMC10853229; doi:10.1038/s41598-024-53551-w)
Supplement: Supplementary file 1 — Supplementary Tables. [file 41598_2024_53551_MOESM1_ESM.docx]

**Supplementary material**

**Supplementary material 1.**

A breakdown of the levels and behaviours displayed in the aggressive survey video.

| **Time (seconds)** | **Distance (start to end of level) (m)** | **Level** | **Dog behaviours displayed** |
| --- | --- | --- | --- |
| 0.0 – 9.7 (9.7) | 0.7m (5–4.3m) | 0 | - Lying down, panting, looking left and right.   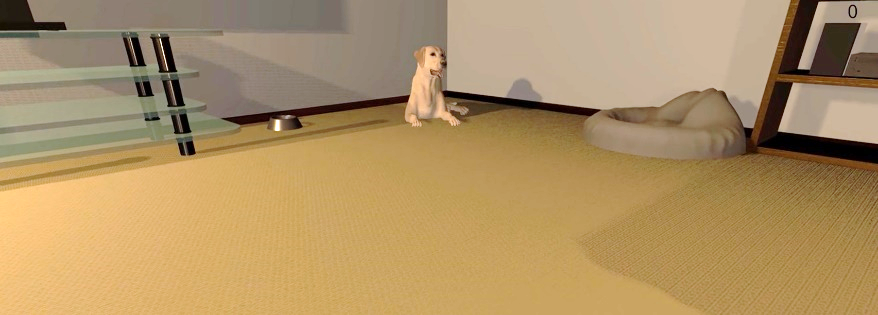 |
| 9.7 – 28.0 (18.3) | 1.4m (4.3-2.9 m) | 1 | - Stands up, put paw forward, lip lick, steps back, slowly wagging tail, steps forward, yawns and lip licks, paw raise.   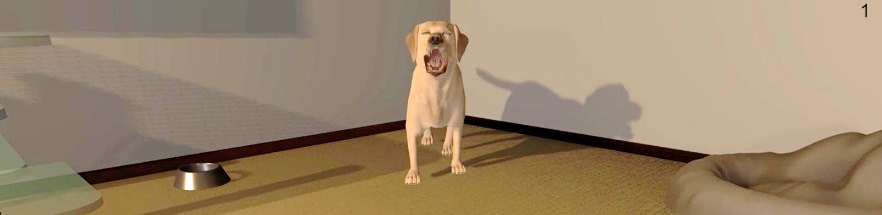 |
| 28.0 – 33.6 (5.6) | 0.4m (2.9-2.5) | 5 | - Head turns with paw raise, head turn with whites of the eye showing (whale eye). Tail now under body.   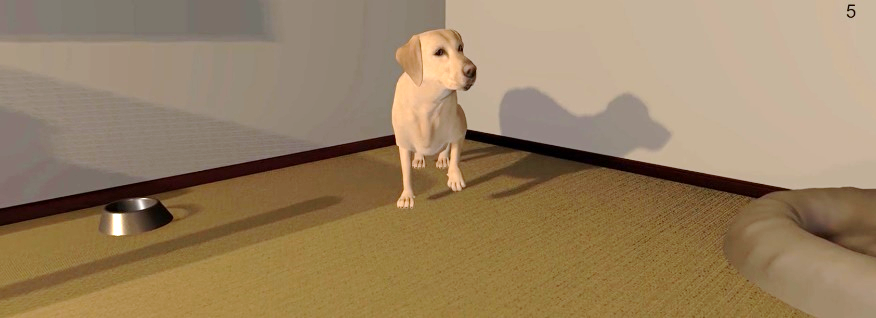 |
| 33.6 - 45.3 (11.7) | 0.9 (2.5 - 1.6) | 6 | - Slowly moves backwards whilst growling and direct eye contact, ears move back and then stands still.   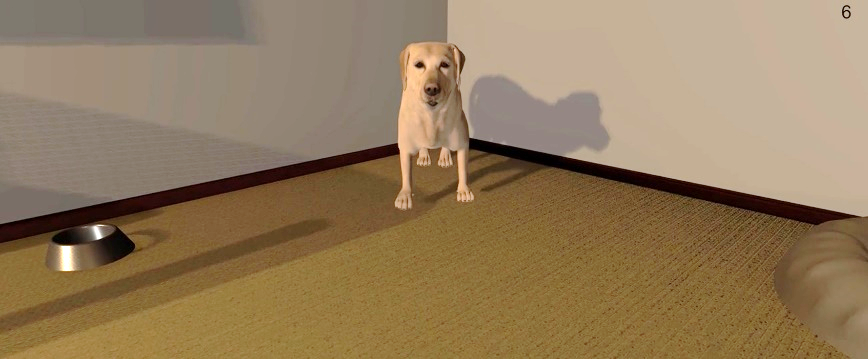 |
| 45.3 - 52.3 (7.0) | 0.5 (1.6 – 1.1) | 7 | - Crouches slightly, barks, direct eye contact, ears back, growling, mouth slightly open with teeth showing.   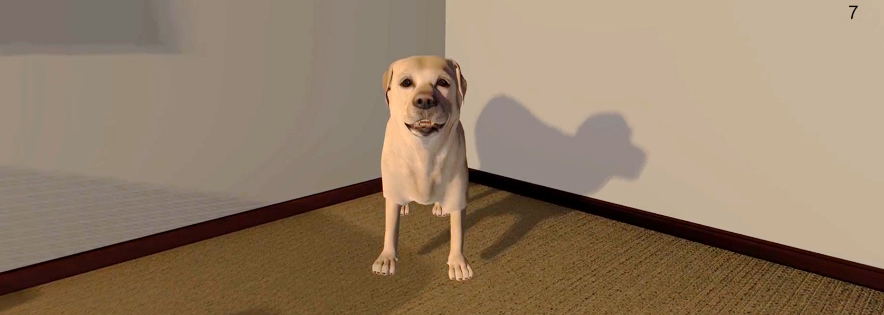 |
| 52.3 – 56.6 (4.3) | 0.3 (1.1 – 0.8) | 8 | - Fully crouching, growling, clench teeth showing, eyes widened with whites of the eye visible, lip lick, growling.   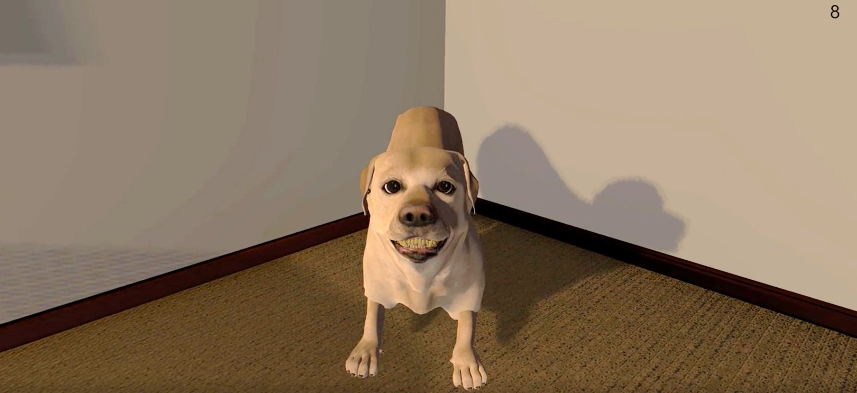 |

**Supplementary material 2.**

Responses to six dog-related statements for survey 1 and survey 2.

**Survey 1**

| **Statement** | **SD** | **%** | **D** | **%** | **N** | **%** | **A** | **%** | **SA** | **%** | **Total** |
| --- | --- | --- | --- | --- | --- | --- | --- | --- | --- | --- | --- |
| 1. "I am cautious in the presence of most dogs” | 10 | 25 | 13 | 32.5 | 8 | 20 | 8 | 20 | 1 | 2.5 | 40 |
| 2. “I enjoy the presence of most dogs” | 0 | 0 | 0 | 0 | 0 | 0 | 7 | 17.5 | 33 | 82.5 | 40 |
| 3. “I feel relaxed in the presence of most dogs” | 0 | 0 | 0 | 0 | 1 | 2.5 | 16 | 40 | 23 | 57.5 | 40 |
| 4. "I can recognise when a dog is showing aggressive behaviours” | 0 | 0 | 0 | 0 | 2 | 5 | 27 | 67.5 | 11 | 27.5 | 40 |
| 5. “I can recognise when a dog is showing scared/fearful behaviours” | 0 | 0 | 0 | 0 | 2 | 5 | 32 | 80 | 6 | 15 | 40 |
| 6. “I can recognise when a dog is showing relaxed behaviours” | 0 | 0 | 0 | 0 | 1 | 2.5 | 28 | 70 | 11 | 27.5 | 40 |

**Survey 2**

| **Statement** | **SD** | **%** | **D** | **%** | **N** | **%** | **A** | **%** | **SA** | **%** | **Total** |
| --- | --- | --- | --- | --- | --- | --- | --- | --- | --- | --- | --- |
| 1. "I am cautious in the presence of most dogs” | 7 | 17.5 | 14 | 35 | 10 | 25 | 8 | 20 | 1 | 2.5 | 40 |
| 2. “I enjoy the presence of most dogs” | 0 | 0 | 0 | 0 | 0 | 0 | 7 | 17.5 | 33 | 82.5 | 40 |
| 3. “I feel relaxed in the presence of most dogs” | 0 | 0 | 0 | 0 | 0 | 0 | 18 | 45 | 22 | 55 | 40 |
| 4. "I can recognise when a dog is showing aggressive behaviours” | 0 | 0 | 0 | 0 | 0 | 0 | 30 | 75 | 10 | 25 | 40 |
| 5. “I can recognise when a dog is showing scared/fearful behaviours” | 0 | 0 | 0 | 0 | 1 | 2.5 | 31 | 77.5 | 8 | 20 | 40 |
| 6. “I can recognise when a dog is showing relaxed behaviours” | 0 | 0 | 0 | 0 | 1 | 2.5 | 30 | 75 | 9 | 22.5 | 40 |

**Supplementary material 3.** Participants’ agreement with statements regarding the behaviour and appearance of the virtual dog compared to a real dog.

|  | **Survey 1** | | **Survey 2** | |
| --- | --- | --- | --- | --- |
|  | **Non-reactive** | **Aggressive** | **Non-reactive** | **Aggressive** |
| ***“The dog’s behaviour in the video was similar to that of a real dog”*** | n (%) | n (%) | n (%) | n (%) |
| Strongly Agree | 7 (17.5) | 7 (18.4) | 5 (12.8) | 7 (17.5) |
| Agree | 26 (65.0) | 27 (71.1) | 29 (74.4) | 29 (72.5) |
| Neither agree/disagree | 7 (17.5) | 4 (10.5) | 4 (10.3) | 4 (10.0) |
| Disagree | 0 | 0 | 1 (2.6) | 0 |
| Strongly Disagree | 0 | 0 | 0 | 0 |
| Total | 40 (100.0) | 38 (100.0) | 39 (100.0) | 40 (100.0) |
| Missing | 0 | 2 | 1 | 0 |
| ***“The appearance of the dog in the video was similar to that of a real dog”*** |  |  |  |  |
| Strongly Agree | 9 (23.7) | 9 (22.5) | 5 (13.5) | 5 (12.8) |
| Agree | 22 (57.9) | 23 (57.5) | 27 (73.0) | 29 (74.4) |
| Neither agree/disagree | 6 (15.8) | 8 (20.0) | 5 (13.5) | 5 (12.8) |
| Disagree | 1 (2.6) | 0 | 0 | 0 |
| Strongly Disagree | 0 | 0 | 0 | 0 |
| Total | 38 (100.0) | 40 (100.0) | 37 (100.0) | 39 (100.0) |
| Missing | 2 | 0 | 3 | 1 |

**Supplementary material 4.** Veterinary student responses to the question “*Currently how confident do you currently feel in your ability to interpret canine behaviour?*”. Ratings: 1 = Not at all, 2 = Slightly confident. 3 = Somewhat confident, 4 = Fairly confident, 5 = Very confident.

|  | **Control**  **Pre-intervention 1**  **(n = 23)** | | **Control**  **Pre-intervention 2**  **(n = 23)** | | **Intervention Pre-intervention 1**  **(n = 17)** | | **Intervention**  **Post-intervention 1**  **(n = 17)** | |
| --- | --- | --- | --- | --- | --- | --- | --- | --- |
| **Answer** | n (%) | Mean (median)  (1-5) | n (%) | Mean (median) (1-5) | n (%) | Mean (median)  (1-5) | n (%) | Mean (median)  (1-5) |
| Not at all confident | 0 (0) | 3.26 (3.00) | 0 | 3.78 (4.00) | 1 (5.9) | 3.18 (3.00) | 0 | 3.88 (4.00) |
| Slightly confident | 5 (21.7) |  | 0 |  | 2 (11.7) |  | 0 |  |
| Somewhat confident | 9 (39.1) |  | 5 (21.7) |  | 7 (41.2) |  | 4 (23.5) |  |
| Fairly confident | 7 (30.4) |  | 18 (78.3) |  | 7 (41.2) |  | 11 (64.7 |  |
| Very confident | 2 (8.7) |  | 0 |  | 0 |  | 2 (11.8) |  |

The intervention survey group (n = 17) combined with an additional eight students who completed the first, second, intervention and third surveys from the control group (n = 25). Ratings: 1 = Not at all, 2 = Slightly confident, 3 = Somewhat confident, 4 = Fairly confident, 5 = Very confident.

| **Answer** | **Pre-survey***  **n (%)**  **(n = 25)** | **Mean (median)**  **(1-5)** | **Post-survey**  **n (%)**  **(n = 25)** | **Mean (median) (1-5)** | **Wilcoxon, P value** |
| --- | --- | --- | --- | --- | --- |
| Not at all confident | 1 (4.0) | 3.16 (3.00) | 0 | 3.92 (4.00) | Z = -4.146 P <0.001 |
| Slightly confident | 4 (16.0) |  | 0 |  |  |
| Somewhat confident | 10 (40) |  | 5 (20) |  |  |
| Fairly confident | 10 (40) |  | 17 (68) |  |  |
| Very confident | 0 |  | 3 (12) |  |  |

*Control group participants (n = 8) pre-survey data was from the first survey (survey 1).

A breakdown and comparison of the ‘combined group’ (n =25) including survey 3 of the control group (n = 8) and survey 2 of the intervention group (n = 17).

| **Answer** | **Control**  **Survey 3**  **n (%)**  **(n = 8)** | **Mean (median)**  **(1-5)** | **Intervention**  **Survey 2**  **n (%)**  **(n = 17)** | **Mean (median) (1-5)** |
| --- | --- | --- | --- | --- |
| Not at all confident | 0 | 4 .00 (4.00) | 0 | 3.88 (4.00) |
| Slightly confident | 0 |  | 0 |  |
| Somewhat confident | 1 (12.5) |  | 4 (23.6) |  |
| Fairly confident | 6 (75.0) |  | 11 (64.7) |  |
| Very confident | 1 (12.5) |  | 2 (11.8) |  |

**Supplementary material 5.** Behaviours seen in the full-length aggressive video task. *Only participants were included that completed the questions for both survey 1 and survey 2 in the control (n = 20) and intervention (n =16) group.

|  | **Survey 1 - Control (n = 20)** | | | | **Survey 2 - Control (n = 20)** | | | | **McNemar’s Test** |
| --- | --- | --- | --- | --- | --- | --- | --- | --- | --- |
| **Behaviours** | **Yes** | **No** | **Total** | **Yes** | | **No** | **Total** | **P value** | |
| Lip lick | 15 (75) | 5 (25) | 20 | 19 (95) | | 1 (5) | 20 | 0.219 | |
| Yawn | 16 (80) | 4 (20) | 20 | 15 (75) | | 5 (25) | 20 | 0.625 | |
| Paw raise | 13 (65) | 7 (35) | 20 | 18 (90) | | 2 (10) | 20 | 0.754 | |
| Head turn | 15 (75) | 5 (25) | 20 | 19 (95) | | 1 (5) | 20 | 0.219 | |
| Backing away | 20 (100) | 0 (0) | 20 | 20 (100) | | 0 (0) | 20 | - | |
| Show teeth | 20 (100) | 0 (0) | 20 | 20 (100) | | 0 (0) | 20 | - | |
|  | | | | | | | | | |
|  | **Survey 1 - Int. (n = 16)** | | | | **Survey 2 – Int. (n = 16)** | | | | **McNemar’s Test** |
| **Behaviours** | **Yes** | **No** | **Total** | **Yes** | | **No** | **Total** | **P value** | |
| Lip lick | 8 (50) | 8 (50) | 16 | 14 (87.5) | | 2 (12.5) | 16 | **0.031** | |
| Yawn | 15 (93.7) | 1 (6.3) | 16 | 15 (93.7) | | 1 (6.3) | 16 | 1.000 | |
| Paw raise | 15 (93.7) | 1 (6.3) | 16 | 16 (100) | | 0 | 16 | 1.000 | |
| Head turn | 12 (75) | 4 (25) | 16 | 14 (87.5) | | 2 (12.5) | 16 | 0.625 | |
| Backing away | 16 (100) | 0 (0) | 16 | 16 (100) | | 0 | 16 | - | |
| Show teeth | 16 (100) | 0 (0) | 16 | 16 (100) | | 0 | 16 | - | |

**Supplementary material 6.** Perceived meaning of the lip lick behaviour in the aggressive video task (open ended questions)

| **Lip lick - Survey 1 Control (n = 20)** |  |  | **Lip lick - Survey 2 Control (n = 20)** |  |
| --- | --- | --- | --- | --- |
| **Description** | **n** |  | **Description** | **n** |
| Emotion/feeling |  |  | Emotion/feeling |  |
| Anxiety/Anxious | 9 |  | Anxiety/Anxious | 9 |
| Stress(ed) | 7 |  | Stress(ed) | 3 |
| Scared/Fearful | 6 |  | Scared/Fearful | 3 |
| Nervous | 3 |  | Uncomfortable, discomfort | 2 |
| Worried | 2 |  | Nervous | 1 |
| Uncomfortable | 1 |  | Worried | 1 |
| Leave alone/do not come closer/ stop | 4 |  | Submissive | 1 |
| Preparing to/occurs before an attack/bite | 2 |  | Threatened | 1 |
| Due to increased or decreased salivation | 2 |  | Due to increased or decreased salivation | 2 |
| Bottom of Ladder of Aggression | 1 |  | Preparing to/occurs before an attack/bite | 1 |
| Preparing to fight/flight | 1 |  | Bottom of Ladder of Aggression | 1 |
| They (dog) are not a threat | 1 |  | Preparing to fight/flight | 1 |
|  |  |  | They (dog) are not a threat, avoid confrontation | 1 |
|  |  |  | Hungry | 1 |
|  |  |  | A warning that they can bite/have teeth | 1 |
|  |  |  |  |  |
| **Lip lick - Survey 1 Int. (n = 16)** |  |  | **Lip lick - Survey 2 Int. (n =16)** |  |
| **Description** | **n** |  | **Description** | **n** |
| Emotion/feeling |  |  | Emotion/feeling |  |
| Anxiety/Anxious | 4 |  | Anxiety/Anxious | 8 |
| Scared/Fearful | 3 |  | Scared/Fearful | 3 |
| Stress | 2 |  | Stress | 2 |
| Nervous | 2 |  | Nervous | 3 |
| Uncomfortable | 2 |  | Worried | 1 |
| Uneasy, unsure, on edge | 2 |  | Aggressive/anger | 1 |
| Confusion | 1 |  | Trying to get the approach to stop | 1 |
| Defensive | 1 |  |  |  |
| Warning signs of aggression/defensive | 1 |  |  |  |
| Hungry | 1 |  |  |  |
| Leave alone, do not come closer, stop | 1 |  |  |  |
| Ready to attack/bite | 1 |  |  |  |

**Supplementary material 7.** Perceived meaning of the yawn behaviour in the aggressive video task (open ended question)

| **Yawn - Survey 1 Control (n = 20)** |  |  | **Yawn - Survey 2 Control (n = 20)** |  |
| --- | --- | --- | --- | --- |
| **Description** | **n** |  | **Description** | **n** |
| Emotion/feeling |  |  | Emotion/feeling |  |
| Stressed | 10 |  | Stressed | 7 |
| Anxious | 9 |  | Anxious | 9 |
| Scared/fearful | 2 |  | Scared/fearful | 3 |
| Relaxed | 1 |  | Relaxed | 1 |
| Tired | 1 |  | Nervous | 1 |
| Discomfort | 3 |  | Tired / sleepy | 1 |
| Comfortable | 2 |  | Feels threatened | 1 |
| It is not a threat | 1 |  | Worried | 2 |
| Fight or flight response | 1 |  | Discomfort/Uncomfortable | 1 |
| Showing weakness | 1 |  | Try to warn human off / Does not want to be approached | 2 |
|  |  |  | A sign of aggression | 1 |
|  |  |  | Submission | 1 |
|  |  |  | Calm itself / self soothe | 1 |
|  |  |  | Fight or flight response | 1 |
|  |  |  | I don't know | 2 |
|  |  |  |  |  |
| **Yawn - Survey 1 Int. (n = 16)** |  |  | **Yawn - Survey 2 Int. (n = 16)** |  |
| **Description** | **n** |  | **Description** | **n** |
| Emotion/feeling |  |  | Emotion/feeling |  |
| Anxious | 4 |  | Anxious | 5 |
| Stressed | 3 |  | Stressed | 3 |
| Relaxed | 2 |  | Scared/fearful | 3 |
| Tired | 2 |  | Nervous | 3 |
| Nervous | 1 |  | Unsure | 2 |
| Confused | 1 |  | Anger | 1 |
| Warning | 2 |  | Warning to back away | 2 |
| Unease | 2 |  | High behavioural arousal | 1 |
| Showing teeth | 2 |  | Patience is running out | 1 |
| Ladder of aggression | 1 |  | I don't know | 1 |
| Sign of appeasement | 1 |  |  |  |
| I don't know | 3 |  |  |  |

**Supplementary material 8**. Perceived meaning of the paw raise behaviour in the aggressive video task (open ended question).

| **Paw raise - Survey 1 Control (n = 20)** |  |  | **Paw raise - Survey 2 Control (n = 20)** |  |
| --- | --- | --- | --- | --- |
| **Description** | **n** |  | **Description** | **n** |
| Emotion/feeling |  |  | Emotion/feeling |  |
| Anxious | 3 |  | Anxious | 5 |
| Uncertain | 4 |  | Uncertain / Uneasy | 6 |
| Scared/fearful | 4 |  | Scared/fearful | 3 |
| Nervous | 1 |  | Nervous | 2 |
| Uncomfortable | 1 |  | Relaxed | 1 |
| Preparing to move / back away | 8 |  | Feels threatened | 2 |
| Preparing to warn/lunge /attack | 2 |  | Preparing to move / back away | 6 |
| Vulnerable | 1 |  | Trying to hide his fear | 1 |
| Moving up the Ladder of Aggression | 1 |  | Vulnerable | 1 |
| Lip lick not working, so trying other signs | 1 |  | Submissive | 2 |
| Submissive | 1 |  | I don't know | 2 |
| I don't know | 4 |  |  |  |
|  |  |  |  |  |
| **Paw raise - Survey 1 Int. (n = 16)** |  |  | **Paw raise - Survey 2 Int. (n = 16)** |  |
| **Description** | **n** |  | **Description** | **n** |
| Emotion/feeling |  |  | Emotion/feeling |  |
| Anxious / agitated | 2 |  | Anxious | 3 |
| Uncertain / uneasy | 3 |  | Uncertain/uneasy | 3 |
| Scared/fearful | 3 |  | Scared/fearful | 2 |
| Nervous | 1 |  | Uncomfortable | 2 |
| Feeling trapped | 1 |  | Angry | 1 |
| Fight or flight | 3 |  | Nervous | 1 |
| Preparing to move / back away | 1 |  | Stressed | 1 |
| Confrontational / Trying to show power | 2 |  | Submissive | 4 |
| Concentrating | 1 |  | Preparing to move / back away | 3 |
| Anticipating a stressor | 1 |  | Vulnerable | 2 |
| I don't know | 1 |  | Appeasement to get person to stop / back off / dog wants it’s space | 2 |
|  |  |  | Alert and watching | 1 |
|  |  |  | I don't know | 1 |

**Supplementary material 9**. Mean and median Igroup presence ratings across control and intervention groups.

|  | **Control (Survey 1)** | | **Control (Survey 2)** | |  | **Intervention (Survey 1)** | | **Intervention (Survey 2)** | |  |
| --- | --- | --- | --- | --- | --- | --- | --- | --- | --- | --- |
|  | Mean | Median | Mean | Median | P | Mean | Median | Mean | Median | P |
| General Presence | 4 | 4 | 3.55 | 4 | 0.171 | 4.06 | 4 | 3.81 | 4 | 0.271 |
| Spatial Presence | 3.19 | 3 | 3.25 | 3 | 0.948 | 2.99 | 3 | 3.11 | 3 | 0.439 |
| Involvement | 2.88 | 3 | 2.91 | 3 | 0.737 | 2.84 | 3 | 2.73 | 2 | 0.671 |
| Experienced Realism | 2.71 | 3 | 2.79 | 3 | 0.602 | 2.59 | 3 | 2.67 | 3 | 0.648 |
